# The barriers and facilitators of herpes zoster vaccination intentions of urban residents in China: a qualitative study

**DOI:** 10.1186/s41256-025-00413-1

**Published:** 2025-04-18

**Authors:** Beibei Yuan, Chao Long, Ming Wang, Elizabeth Maitland, Stephen Nicholas, Xianjing Qin, Weiying Zhao, Dawei Zhu, Ping He

**Affiliations:** 1 China Center for Health Development Studies, Peking University, Beijing, China; 2 School of Public Health, Peking University, Beijing, China; 3 School of Management, University of Liverpool, Liverpool, UK; 4 Health Services Research and Workforce Innovation Centre, Newcastle Business School, University of Newcastle, Newcastle, NSW Australia; 5 Australian National Institute of Management and Commerce, 1 Central Avenue Australian Technology Park, Sydney, Australia; 6 Health Policy Research Center, Guangxi Medical University, Nanning, China; 7 School of Medicine, Shaoxing University, Shaoxing, Zhejiang China; 8 Department of Pharmacy Administration and Clinical Pharmacy, School of Pharmaceutical Sciences, Peking University, Beijing, China; 9 International Research Center for Medicinal Administration (IRCMA), Peking University, Beijing, China

**Keywords:** Barriers, Facilitators, Herpes zoster vaccinate, Vaccine hesitancy, Qualitative study

## Abstract

**Background:**

In an aging society, herpes zoster (HZ) increases the health burden on infected patients. While quantitative studies point to a lack of willingness to accept the HZ vaccine in China, there is limited number of studies with in-depth qualitative analysis on HZ vaccination intention. This study undertakes a qualitive study method to identify the barriers and facilitators behind urban residents’ HZ vaccination intention in three China cities, and contributes towards some targeted vaccination promotion suggestions to China and other LMICs with similar low coverage of HZ vaccination.

**Methods:**

We conducted 12 focus group discussions in three cities of China. In each discussion we recruited 3 to 6 participants aged 20 and older to catch the views on the HZ vaccine from residents with a wider age range. Participants were recruited by purposive sampling techniques. Guided by the health belief model, thematic analysis was used to group participants’ HZ vaccine attitudes and to identify the barriers and facilitators to HZ vaccination.

**Results:**

The attitude of 59 participants participating in the focus group discussions showed a low-level acceptability of the HZ vaccine with only 27.1% (16/59) displaying a willingness to HZ vaccine uptake. The barriers to HZ vaccination included limited or incorrect conception on HZ prevalence, risk factors, susceptibility, symptoms, prevention and treatment methods, and the high cost of the HZ vaccine. Perceived vulnerability to HZ, fear of HZ pain and individuals' financial capacity were the strongest facilitators to HZ vaccination. In addition, it was found that advocacy of HZ vaccination by health professionals or government financial subsidies to HZ vaccination, could attenuate the above barriers to HZ vaccine uptake.

**Conclusions:**

Our study revealed a series of barriers and facilitators of HZ vaccination intention. We recommend HZ education and advocacy by health workers and government health officials to address the limited HZ knowledge and HZ misconceptions, and the government (or health insurance providers) to pay or subsidize the high costs of HZ vaccination to increase the HZ vaccination rate.

## Background

Herpes zoster (HZ) is a condition caused by latent varicella zoster virus reactivation. The global incidence of HZ ranges between 3 and 5 per 1,000 person-years [1], with persons living to 85 years old having a 50% risk of HZ in the absence of the HZ vaccine [2]. While individuals of any age can be at risk of being HZ infected, studies show that the incidence of HZ increases with age, posing a specific health risk to China’s aging population [3]. Patients with HZ mostly experience localized and unilateral eruption of grouped vesicles on an erythematous base, general malaise and usually mild-to-moderate pain in the area of the affected dermatome, all of which reduce the patients’ normal functioning and quality of life [4]. There are several complicated disease courses like post-herpetic neuralgia (PHN) with pain persisting for more than three months, and neurological complications including transient segmental paralysis which may result in abdominal wall hernias and bladder dysfunction, as well as encephalitis and meningitis [5]. A US study found that HZ and its complications caused the loss of approximately 67,000 quality-adjusted life years (QALYs) and incurred costs of US$2.4 billion in direct medical costs and productivity losses annually.[6].

The treatments for HZ include antiviral therapy, which reduces the risk of post-herpetic neuralgia; systemic analgesics for controlling pain based on assessment outcomes of pain intensity scale; and topical treatment of skin lesions [7]. As a prevention intervention, the varicella zoster vaccine has been available since 1970s, with the attenuated live vaccine Zostavax® used in adults aged 60 and older since 2006 [8]. A large-scale study of Zostavax® in 2008 reported a reduced incidence of HZ by 51.3% and PHN by 66.5% [8], with only mild injection site reactions being noted as adverse effects. A novel recombinant HZ vaccine Shingrix® was approved in 2017, and the approval study showed that vaccine was effective in more than 90% of cases with respect to HZ and in more than 89% of cases with respect to chronic pain and PHN [9]. Compared with the treatments for HZ, HZ vaccination provided a cost-effective way to improve older adults’ quality of life [10], so routine vaccination is widely recommended for people aged 60 and older. Published data in 2018 showed that the coverage of HZ vaccine in those aged 70 and older was 41% in US [11], 56% in UK [12] and 47% in Australia [13]. The coverage data in low- and middle-income countries (LMICs) were limited and showed that the coverage in those aged 50 and older was only 3.7% in United Arab Emirates [14] and 2.8% in Hong Kong, China [15].

In China, data from 24 hospitals in seven cities showed a HZ prevalence rate of 7.7%, with at least 2.77 million yearly HZ cases [16]. Based on limited survey data from five Chinese towns, the cumulative incidence of HZ among people aged 80 and older was 3.34 times that of those aged 50 and older, and HZ imposed an average direct treatment cost per patient between US$56—US$251 with a 4.53% hospitalization rate and the mean duration of hospitalization between 8.44 and 13.87 days [16]. Since 2019, Shingrix® is the only approved HZ vaccine for use in China. One online survey in 2022 showed that the coverage in those aged 50 and older was 10.34% [17] and several studies in specific cities and provinces pointed to a lack of HZ vaccine knowledge and lack of willingness to accept the vaccine [17, 18]. Given HZ vaccine being a cost-effective intervention for improving the quality of life of the vaccinated [19] and China’s aging population bringing the predicted rapidly increasing disease burden from HZ, it is necessary to promote the vaccination coverage.

Understanding of the determinants of HZ vaccination intention is the first step towards promotion strategies. Existing HZ researches in China are quantitative studies, analyzing the influencing factors on the HZ vaccination intention using univariate or multivariate analysis [20, 21]. Qualitative studies related to perceptions of vaccination choices and the factors that drive adults to vaccination (or not) were mainly conducted in high-income countries [22–25]. The qualitative studies providing an in-depth understanding of the views of adults towards the HZ vaccine in China and other LMICs are still lacking. This study undertakes a qualitative focus group approach to assess the perception of Chinese adults towards the HZ vaccine and identify the facilitators and barriers to the HZ vaccination intention. This study could contribute towards more targeted vaccination promotion strategies in China and other LMICs with similar financial constrains in expanding national immunization program and low coverage of HZ vaccination, through supplementing findings from quantitative studies in China and qualitative studies in countries with very different settings.

## Methods

### Study design

This is a qualitative study following an interpretivist research paradigm, where participant views are subjective, contextual and socially constructed [26]. The qualitative data were collected using focus group discussions with adults aged 20 or older in three diverse Chinese cities. The data collection and analysis were guided by phenomenology approach, evaluating the tendency of participants to HZ vaccination from their expressed attitudes and extracting the factors forming the vaccination attitudes from their experiences and feelings.

### Sampling

To capture views representative of different socio-economic contexts, the sample cities were selected from different geographical regions of China based on different economic development levels: Beijing in the north of China was a highly-developed economic city; Weifang in eastern China had a middle-developed economic level; and Nanning was in the less developed agricultural southwest Guangxi autonomous region. Purposive sampling was adopted in each city, with four communities in different sub-city districts with different levels of social and economic development selected on the recommendation of the local health administration department. Although the HZ vaccine at market mainly targets the people aged 50 and older, we recruited participants over 20 years old to catch a wider cohort of adults’ views on the HZ vaccine, especially development of HZ vaccines in future may cover wider age range, at the same time in China young adults usually play an important role in the health management of their older parents. Supported by the community health centers, the study tried to recruit two residents aged 20 to 40 years old, two residents aged 40 to 60 and two residents aged over 60 in each community with a combination of volunteer and snowball sampling. Among the recruited participants, the study also tried to include participants with a chicken pox disease history, HZ disease history or PHN 1 disease history. Not all community health centers could recruit the target number of participants with these characteristics, so the final sample for each focus group discussion ranged between 3 to 6 participants.

### Data collection method

Between October 15–30, 2022, qualitative data were collected during focus group discussions based around questions co-developed by the research team. Once participants were recruited, the lead researcher established rapport with participants and conducted the group discussion meetings, which encouraged open discussion, with participant interaction and dialogue allowing the interchange of ideas and comments on each participant’s points of view [27]. During the discussion, the lead researcher encouraged each participant to talk about their feelings and opinions to avoid dominant participants leading or guiding the discussion.

The research team developed questions that structured the focus group discussion, comprising a series of open-ended questions about participants’ knowledge on HZ and the HZ vaccine; attitudes and beliefs about the HZ vaccine; their willingness to HZ vaccination; willingness to support their parents’ HZ vaccination; the reasons for HZ vaccine hesitancy; their attitude to current HZ vaccine prices; and their willingness to pay for the HZ vaccine. The interview guide can be found in the Supplement. For those participants who had no knowledge of HZ and the HZ vaccine, we conducted a guided open-ended discussion, asking participants about their previous experiences of purchasing vaccines for themselves and their family, including parents, and their general opinion on purchasing vaccines to prevent disease. As new topics emerged during the discussions, the lead researcher probed the detailed experiences or ideas of the participants. The focus group discussion ended when the structured questions were exhausted and there were no new ideas emerging. Each focus discussion lasted 45–90 min, and all the discussions were in Mandarin. Participants received a gift valued about RMB20 for their participation.

All the focus group discussions were audio-recorded and transcribed verbatim. In the transcripts, the participants were labeled by an identification number as city (Beijing: BJ, Weifang: WF, Nanning: NN)_district (Chaoyang and Daxing in Beijing: CC and DX; Nanguan, Dayu, Kuiwen and Xiguan in Weifang: NG, DY, KW and XG; Jiangnan, Qingxiu and Liangqing in Nanning: JN, QX and LQ)_gender (Female: F, Male: M)_age. The research team processed the primary data within two days of each focus group discussion, ensuring that the last focus group discussion reached the saturation point of the descriptive themes. The study received human subject ethics approval from Peking University and participants gave informed consent to be interviewed and recorded.

### Data processing and analysis

The data were analyzed using thematic analysis. We combined deductive and inductive approaches. Guided by the health belief model (HBM), a theoretical model widely used in analysis on preventive health behaviors [28] and to guide health promotion and disease prevention programs [29], the analysis used deductive approaches to identify and group the key themes from the data. According to the HBM, individuals’ health behavior changes to prevent diseases based on their state of psychological willingness [30, 31]. In the psychological vaccine decision-making process, vaccination behaviors are conceived as the output of an individual’s perceptions about a disease and its related vaccine, including the perceived severity and susceptibility to the disease and the perceived benefits and risks of the vaccine. Second, our analysis also used inductive approach to extract original themes emerging from the discussion. During the process of coding and analysis, we also grouped the participants into a positive attitude group and negative/unclear attitude group based upon participants’ expressions on their willingness to accept HZ vaccination. As different themes were extracted from groups with different intention, barriers or facilitators to vaccination were confirmed.

The coding process was in Mandarin. Three researchers conducted the analysis, comprising one associate professor, one PhD student and one master student all with formal qualitative study method training and previous experience in practicing qualitative health research. In addition, the three researchers were all trained in health science research. Three researchers separately read 10 transcripts each, formed initial impressions of the data, and coded the distinct ideas as descriptive themes; the descriptive themes were then further refined and grouped into analytical themes using HBM; then all researchers discussed the emergent themes together, reviewed the transcripts and further refined the themes. After several rounds of separate analysis and group discussions, the research team finished the thematic extraction until no new themes emerged; sorted and categorized all the themes based on whether they were related to benefits or costs, disease or vaccine; and then looked for the patterns within and across the vaccine willingness and vaccine unwillingness (hesitancy and unclear) groups. For example, a participant during the group discussion mentioned that “*we just accept what they (community/government policy) arrange for us to be vaccinated.*”, which was first coded as “trust in government” as a descriptive theme. After discussions among all the researchers and through comparison with “benefits or costs, disease or vaccine” defined in HBM, the “trust in government” theme was then coded as the analytical theme “advocate by authority” that implied endorsement of government could reduce the perceived risk/cost of being vaccinated. When there were no disagreements on the themes and categories among the researchers, the lead researcher confirmed the themes and sub-themes and returned to the transcripts to identify exemplar quotes if necessary. The data processing and analysis were conducted from October 17 to November 10, 2022.

All the analysis were conducted in NVivo12 software, and when writing the manuscript the exemplar quotes were translated into English by a research assistant with English major undergraduate and checked by the associate professor in analysis team.

## Results

### Study sample and HZ vaccination intention

Twelve focus group discussions were conducted with each group having 3 to 6 participants. Table 1 displays information on the 59 adults interviewed, including data on sex, age and geographic region. There were 17 participants from Beijing, 22 from Weifang and 20 from Nanning; 36 were female and 23 are male and the ages ranged from 20 to 80. 28 participants expressed their unwillingness to vaccination; 16 expressed a willingness to vaccination; and 15 did not explicitly expressed their attitude to HZ vaccination. As Table 1 shows, the proportion of the participants with willingness to vaccination was higher in Beijing, in the female group and in the participants aged 40–59.

**Table 1:** The characteristics and attitude to HZ vaccination

Attitude	All (n)	Positive (n, %)	Negative (n, %)	Unclear (n, %)
District				
Beijing	17	5, 29.4	8, 47.1	4, 23.5
Weifang, Shandong	22	6, 27.3	10, 45.5	6, 27.3
Nanning, Guangxi	20	5, 25.0	10, 50.0	5, 25.0
Sex				
Female	36	12, 33.3	16, 44.4	8, 22.2
Male	23	4, 17.4	12, 52.2	7, 30.4
Age				
20–39	12	1, 8.3	7, 58.3	4, 33.3
40–59	28	10, 35.7	9, 32.1	9, 32.1
60 +	19	5, 26.3	12, 63.2	2, 10.5

### Barriers and facilitators to willingness to HZ vaccination

**Fig. 1:**
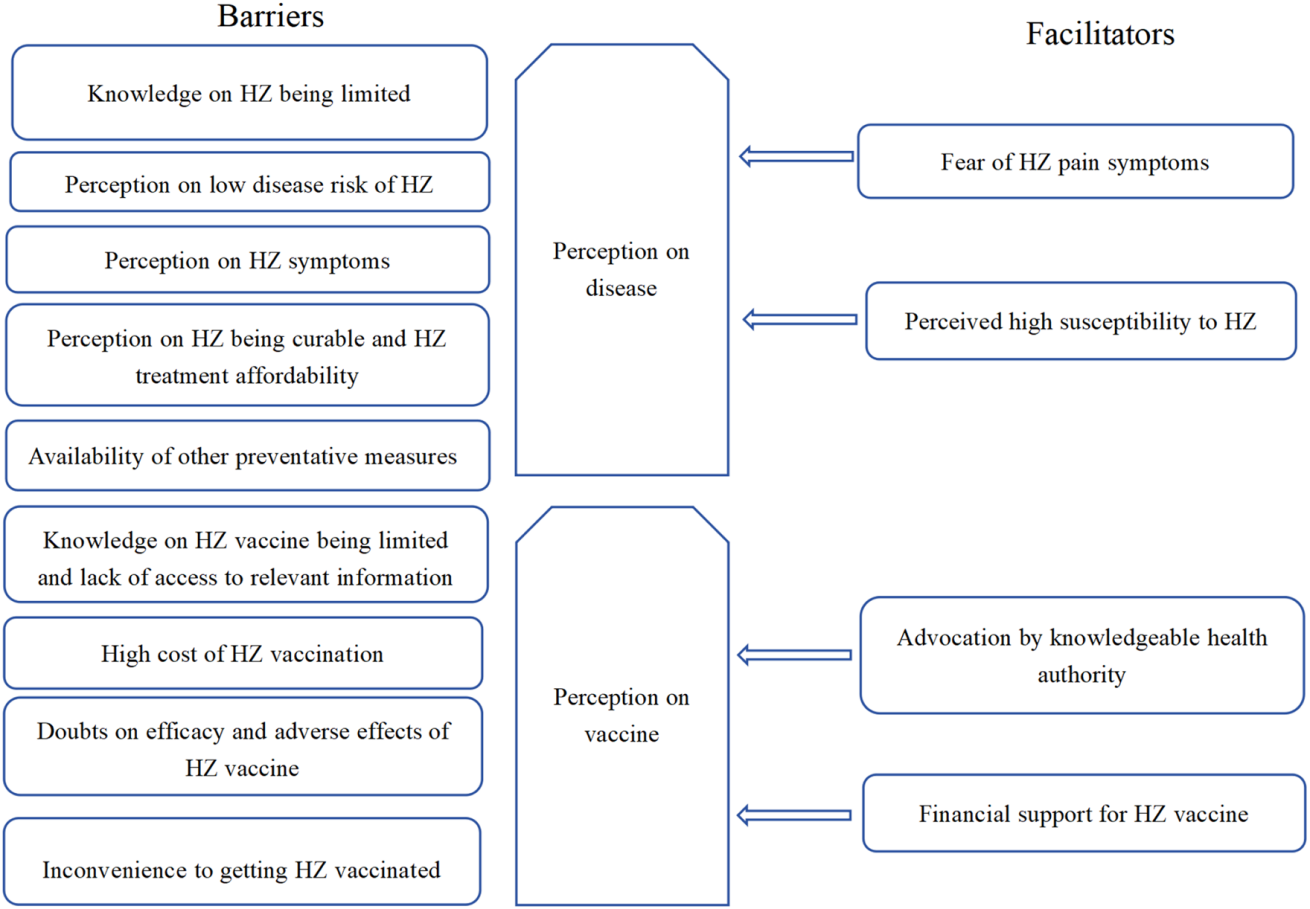
The facilitators and barriers of to HZ vaccination intention

Based on HBM we derived the themes displayed in Fig. 1, and an individual’s decision to vaccination was the output of an individual’s perceptions about the disease and its related vaccine. Transcript analysis yielded five themes related to the perceived severity and susceptibility to the disease (Barrier 1–5) and four themes related to the perceived benefits and risks of the vaccine (Barriers 6–9), most of which were mentioned in those with low willingness to HZ vaccination. Discussants who had an explicit positive attitude to the HZ vaccine, frequently mentioned themes were grouped into different themes as facilitators based on HBM: two themes related to the perceived severity of and high susceptibility to the disease (Facilitator 1–2), two themes related to methods overcoming their doubts on the benefits and risks of the vaccine (Facilitator 3–4).

### Barriers to HZ vaccination

#### Barrier 1: knowledge on HZ, especially limited understanding of the HZ risk factors

All the interviewees expressing a positive HZ vaccination intention had experienced HZ or had relatives experiencing HZ. Roughly half the interviewees in the hesitancy (13/28) or unclear (7/15) groups had never heard of HZ or had superficial knowledge about HZ though the media or social communications. Those in the hesitancy or unclear groups having heard of HZ had only limited information on HZ, usually gained from acquaintances or colleagues mentioning their HZ experiences. Their limited information on HZ, including the symptoms and sequelae, prevalence rate and HZ vulnerable populations, constrained them forming a perception on the severity and susceptibility to HZ. Some participants lacking knowledge on HZ asked the interviewer questions on HZ risk factors, which reflects risk estimation being crucial for health behavior change.

*“[Interviewer: Have you ever heard of the HZ?] No, I haven't. [Interviewer: You don't have any friends around you with HZ?] No, I haven't.” (NN_JN_M_60, 60, Male, Nanning Jiangnan district)–Never heard of HZ*

*“My family members didn't have it. But I do know that shingles seems to happen when there is immune deficiency. And they say it hurts. We never had it in our family. I think one of my colleagues said he got shingles, but I don't know if his shingles formed a ribbon. Oh, maybe I am wrong and his disease was eczema. Is HZ hereditary?” (BJ_DX_F_40, 40, Female, Beijing Daxing district)–Limited knowledge on HZ*

*“Why are people having got chicken pox more likely to get HZ? You should have antibodies against chickenpox? [Interviewer: Yes, it also has antibodies, but you can't get rid of the chickenpox virus. The virus will cause HZ, for example, if your immune system is not good at that time] You mean in case I get old, I might get this virus?” (BJ_DX_F_32, 32, Female, Beijing Daxing district)–Limited knowledge on risk factors*

#### Barrier 2: perception on low disease risk of HZ

Nineteen percent (18.6%; 11/59) of all participants knew about HZ, but thought HZ had a low risk to themselves. This view was especially expressed by younger participants. However, the themes extracted from the interviews revealed the low-risk view was related to the limited number of cases the participants encountered, the fragmentary information on HZ disseminated by health education media, and the incorrect perception of HZ risk factors.

*“I've heard them say that, you know, it's usually one time infection in one life I have got HZ one time, so I might just hold off the vaccination.” (WF_KW_M_63, 63, Male, Weifang Kuiwen district)–Wrong perception on disease risk of those having the history of HZ*

*“I think this (HZ) virus, maybe because I read some information that says we all have it in our bodies. It just happens when you're not well. And this attack, as far as I know, occurs more frequently in people over 80. Young people in their 60s and 70s, after all, have good physical functions and may have low probability to get. At that point, people over 80 might be better (for vaccination)” (BJ_CY_F_54, 54, Female, Beijing Chaoyang district)–Wrong perception on the risks of people aged 60s and 70s age*

*“This thing (HZ vaccination) is like the COVID-19, the vaccine of COVID-19 is free, he/she still has a lot of difficulties (to be vaccinated). Right? I can't. I don't think they'll be convinced (to accept HZ vaccine). And it's not like the flu vaccination that old people know about. Because flu is a common disease. For the elderly, they have flu vaccination every year, because nearly everyone get the general flu every year. They all know that flu is infectious. You see, like flu vaccination, it is hard to get vaccination because of long waiting time, even actually you still get flu after you are vaccinated, and the flu vaccine is not cheap. So I don't think HZ is particularly common.” (BJ_DX_F_40, 40, Female, Beijing Daxing district)–Perception on low HZ prevalence rate compared with the flu*

*“I haven't heard that many people get this disease. It's a rare occurrence.” (NN_QX_M_29, 29, Male, Nanning Qingxiu district)–Perception on low HZ prevalence rate based on personal experience*

#### Barrier 3: past experience with HZ symptoms

About one quarter (23.7%; 14/59) of participants mentioned that they experienced HZ, in which six participants described their symptoms being mild, six described their symptoms being severe with long-term pain, and two did not describe the level of pain. The perception that the HZ impact was mild was related to the belief that HZ vaccination was unnecessary or provided few benefits.

*“When I was a child, I had HZ...I was on grade 3 of junior high school, I felt itch but not pain, and there was a huge red rash,...I heard that one eighth of those having HZ did not feel pain, maybe because I was young at that time.” (WF_KW_F_24, 24, Female, Weifang Kuiwen district)–Past experience of mild symptoms*

*“A little pain, but not too much. Then I had the drip (intravenous injection) for three or five days. [Interviewer: Can you remember whether it was painful?] I'm not in much pain. I just felt itch.” (WF_XG_M_46, 46, Male, Weifang Xiguan district)–Past experience of mild symptoms*

In the six cases of severe HZ pain, only one expressed an unwillingness to be vaccinated, compared to five of the six mild HZ participants’ unwillingness to vaccination.

*“[Interviewer: Does it still hurt now?] No, it doesn’t hurt now. It was very painful for ... the treatment lasted for more than 20 days. I never heard of the vaccine for it. (WF_NG_F_60, 60, Female, Weifang Nanguan district)–Past experience of severe symptoms*

#### Barrier 4: perception on HZ being curable and HZ treatment affordability

All the participants [14] who experienced HZ were cured, even for those participants mentioning HZ cases with serious HZ complications, which resulted in that all participants having experienced HZ, formed the perception that HZ was curable.

*“(I had) few herpes, and (the herpes were) in the waist abdomen. I had infusion (intravenous injection) for 3 days. There was a doctor here that's very convenient, and some patients lined up to get treatment from this doctor, because it's very difficult to treat anywhere else, and there's no magic bullet for this thing (HZ).” (WF_NG_F_52, 52, Female, Weifang Nanguan district)–Past experience of being cured*

During the group discussions, participants often compared the low seriousness of HZ with high fatality diseases, such as cancer or hydrophobia. Six participants mentioned the direct treatment cost for HZ was affordable and significantly lower than HZ vaccine price. Two participants noted that alternative traditional medicine HZ treatments had a very low expense.

*“I got HZ last July...I only took one kind of medicine and spent a little money, about one hundred (RMB Yuan)” (WF_KW_M_63, 63, Male, Weifang Kuiwen district)–Low cost of treatment*

*“It is hard to accept HZ vaccination with current price. Because this disease (HZ) is not universal. The incidence is not that high. In contrast, cervical cancer is cancer and getting will die, but cancer vaccine is not expensive, and the vaccine prevention rate is relatively high.” (WF_KW_F_24, 24, Female, Weifang Kuiwen district)–Not like cancer, HZ being curable*

*“There was a traditional medicine prescription, a medicine made from earthworm, external application on the skin and the skin can recover in several days, and there is no costs.” (WF_XG_M_60, 60, Male, Weifang Xiguan district)–Transitional treatment with low cost*

#### Barrier 5: availability of other preventative measures

Many participants with a low willingness to HZ vaccination preferred alternative HZ preventative measures, such as a healthy diet, physical exercise, staying in a good mood and avoiding stress.

*“It feels HZ like a rare disease. It means low immunity (being the reason of HZ). In general, you should eat and drink healthy food right? ... There should be enough of rest. How to improve immunity, to avoid certain food.... (NN_QX_M_61, 61, Male, Nanning Qingxiu district)–Change in lifestyle to prevent HZ*

*“(I think I will) regulate my mental stress. Because the shot (having injection) hurts. I feel that there must be a virus in my body, because it is often to feel pains, I cannot easily dare to take the vaccine,... I prefer to increasing my resistance (to the virus), do more exercise.” (BJ_CY_F_72, 72, Female, Beijing Chaoyang district)–Change in lifestyle to prevent HZ*

#### Barrier 6: knowledge on HZ vaccine was limited and lack of access to relevant information

A large proportion of participants were not aware of the HZ vaccine. For participants without awareness of the availability of the HZ vaccine, most of them expressed an unwillingness to vaccination when they were informed about the HZ vaccine during group discussions, even for those participants who had experienced HZ or who had heard about serious HZ pain.

*“I have no ideas on this vaccine. (BJ_CY_F_80, 80, Female, Beijing Chaoyang district)–Unawareness on the vaccine*

All the participants who had heard of HZ vaccine lacked specific details, including information on HZ vaccine efficacy and safety. One participant even had the perception that the HZ vaccine was harmful. During the group discussions, participants argued that even health workers they contacted did not have the sufficient information about the HZ vaccine.

*“The advertisements and information on HZ vaccine should be more, we are young and have more channels to get some information, and I think most of people don’t know this vaccine.” (WF_KW_F_40, 40, Female, Weifang Kuiwen District)–Lack of publicity on HZ vaccine*

*“I often hear of HZ vaccine. The vaccine under sale now is not Chinese product, it is imported, and it is expensive. I don’t know the price.” (WF_WC_M_56, 56, Male, Weifang Weicheng District)–Lack of information on HZ vaccine price.*

*“I went to the community health center and have even seen the information on HZ vaccine. Do we need be vaccinated one time every year? ” (NN_QX_M_57, 57, Male, Nanning Qingxiu District)–Lack of vaccine information even in community health center.*

*“[Interviewer: Did the staff in Center for Disease Control say not to get vaccinated?] He/She said it was useless to be vaccinated, because I have been infected by HZ.” (WF_WC_F_56, 56, Female, Weifang Weicheng District)–Health workers having incorrect information on vaccine.*

#### Barrier 7: high cost of HZ vaccination

The price of the HZ vaccine was the most frequently mentioned barrier to vaccination. For the 28 participants expressing vaccine hesitancy, 15 (53.6%) explicitly mentioned the price. The total out-of-pocket cost for a HZ vaccine two dose routine was RMB3200 (USD446), without any insurance coverage or government subsidy. During the discussion, participants measured the financial burden of HZ vaccines by comparing the vaccine costs with their income, HZ treatment costs and the prices of other vaccines. The per capita disposable income for Chinese residents was USD5195.9 in 2023 and the HZ direct treatment costs per patient was USD56-251 based on data in 2019 [32]. Participants’ willingness to pay expressed during discussion ranged from USD14 to 70 for the HZ vaccine, which was considerably lower than the USD446 HZ vaccine price.

*“[Interviewer: What level of prices could you accept?] I think the price should be lower than 500 yuan (USD70)” (NN_QX_F_58, 58, Female, Nanning Qingxiu District)–Big gap between willingness to pay and the actual price*

*“A so-called rich friend of mine has been looking for this vaccine. I don't know if he knows how expensive it is and he has to pay for it.” (BJ_CY_F_65, 65, Female, Beijing Chaoyang District)–Only rich people can afford it.*

*“I think the price for the most of people, like us the retired workers of general private enterprise, not retired staff from government department, we only have not high wages, I think the price is really high. And it only has protection efficacy for several years but not for life. (BJ_CY_F_72, 72, Female, Beijing Chaoyang district)–Compared the wage level and protection efficacy the price being too high*

*“100 yuan (USD7.2) is acceptable. Right? I think it's around 100 (USD7.2)....The treatment of HZ was an oral dose of antiviral medication and a drip (intravenous injection). The western medicine treatment cost a little.” (WF_WC_M_52, 52, Male, Weifang Weicheng district)–Big gap between HZ treatment cost and price of HZ vaccine and prices of other vaccines*

*“Just look at the prices for the children vaccines, and the most expensive vaccine is 200 yuan (USD15), like the varicella vaccine. If HZ vaccine needs so high cost, the gap is too large.” (WF_KW_F_45, 45, Female, Weifang Kuiwen district)–Big gap between HZ price and prices of other vaccines*

During the focus groups, some participants displayed HZ vaccination hesitancy changed to explicit unwillingness to HZ vaccination after being informed the vaccine price being USD446 HZ.

*“If there is vaccine, I must advise them (my parents) to be vaccinated. Yesterday, I watched a video, and it was said almost a third of the people have this kind of thing (HZ)...[Interviewer: The price of one dose is 1600 (USD223) and two doses cost 3200 (USD446)] Compared with my income and other aspects, I have different opinion (on accepting vaccine), I think the government should plan and cover this” (WF_KW_F_60, 60, Female, Weifang Kuiwen district)–Change the positive attitude to HZ vaccine to negative after hearing of price*

#### Barrier 8: doubts on efficacy and adverse effects of HZ vaccine

Shingrix® is effective in ≥ 90% of cases with respect to HZ and in ≥ 89% of cases with respect to chronic pain and PHN [21]. The efficacy of the HZ vaccine was a barrier to vaccination when participants expected the high HZ vaccine price meant near 100% HZ protection.

*“(After hearing of the price of HZ vaccine) My question is how long the vaccine can protect me? If the protection can only last 3 years, I definitely cannot accept this vaccine. If I recommend to my family, I think they will not accept...” (BJ_DX_F_40, 40, Female, Beijing Daxing district)–High price should have better protection efficacy*

*“Can’t it (HZ vaccine) prevent HZ for 100%? [Interviewer: No, it cannot] Too expensive, I cannot afford it.” (WF_WC_M_56, 56, Male, Weifang Weicheng district)–Doubts on efficacy with the high price reducing the willingness to be vaccinated*

Perceptions of the adverse effects of HZ vaccine, including those with chronic diseases, fear of injections and foreign source of the vaccine, were mentioned by discussants as reasons for HZ vaccination hesitancy.

*“My first concern is my health situation. It means what kind of person is not suitable for vaccination, like the current COVID-19 vaccination (not everyone can be vaccinated). For chronic diseases, like coronary heart disease, hypertension and diabetes. So I just have to consider chronic disease (I have), and I'll probably consider HZ vaccine after I get better (from chronic disease).” (BJ_CY_F_72, 72, Female, Beijing Chaoyang district)–Fear of adverse effects*

*“[Interviewer: Why were you parents not vaccinated?] They are afraid of the pain from injection. [Were they worried about the price?] They were not worried about the prices and they have more worries on pains” (WF_KW_F_35, 35, Female, Weifang Kuiwen district)–Fear of pain*

*“I generally consider the vaccine produced by China. [Interviewer: Why] I trust my country and I mainly consider the safety of vaccine.” (NN_JN_F_35, 35, Female, Nanning Jiangnan district)–Preference on the safety of vaccine*

*“I was just vaccinated the flu vaccine at 9th September and 19th September. Generally, I consider the safety problem (especially just be vaccinated the flu vaccine), the vaccine can prevent disease and we still are worried the safety, like if there is clinical trial and other adverse effects.” (NN_QX_M_57, 57, Male, Nanning Qingxiu district)–Fear of the conflict with other vaccines*

#### Barrier 9: inconvenience to getting HZ vaccinated

For participants asking for more information about the HZ vaccine, one barrier was they not knowing what health institutions provide the HZ vaccination.

*“[Interviewer: Do you know where to get vaccination if you want to get vaccinated?] The children go to the Disease Prevention Station (the previous name of County Center for Disease Prevention, not the right place for vaccination). [Interviewer: What about for the elderly]...(no answer)” (WF_KW_F_60, 60, Female, Weifang Kuiwen District)–Lack of information on the providers of HZ vaccine*

Those with knowledge of HZ vaccination institutions or having been vaccinated mentioned that the appointment method and waiting time for vaccine were not user friendly.

*“I ask the China-Japan Friendship Hospital (one private hospital in Beijing), and this hospital did not have HZ vaccine, I want to ask (where can I get HZ vaccine.)” (BJ_CY_F_65, 65, Female, Beijing Chaoyang District)–Lack of information on the providers of HZ vaccine*

*“If I can make an appointment online for vaccination, which may be more convenient. There are many services which cannot be got through online appointments. Unlike the COVID-19 vaccine, which is required to be vaccinated by the government, there is no requirement for other vaccines (including HZ vaccine), but you personally decide (vaccinated or not), so more information is very importance (for making decision)” (NN_QX_F_58, 58, Female, Nanning Qingxiu District)–No convenient channel to get vaccine and need for more information to support decision*

### Facilitators to HZ vaccination

Based on HBM, the analysis found four HZ vaccination facilitators, including two themes related to the perceived HZ severity and high susceptibility (Facilitator 1–2), and two themes related to methods for overcoming HZ doubts on vaccine benefits and risks (Facilitator 3–4).

#### *Facilitator 1: Fear of HZ pain symptoms*

Some participants had knowledge of relatives’ painful experience and negative consequences from HZ, and all had positive attitude to the HZ vaccine or have been vaccinated.

*“I'm retired, 64 years old. I don't say myself, but other friends. I've seen their parents have got HZ, and then they died without treatment.” (BJ_CY_M_64, 64, Male, Beijing Chaoyang District) -- The bad HZ infection experience of residents*

*“My mother got HZ. Then she stayed in hospital for more than 10 days, which was supposed to kill her. Because it hurts too much and it's painful. She couldn't sleep and eat anything. From being infected HZ, the pain had not been stopped, every night she shivered because of pain. The pain was terrible. Afterwards, she came back from the hospital and felt much better, but the hurts still happened exactly at 12:00 p.m. very day. She stayed in pain until she died. It's (The treatment started) just too late” (BJ_DX_F_58, 58, Female, Beijing Daxing District) -- The bad HZ infection experience of residents*

*“I felt pain, and I felt pains every night at 10:30 p.m. and it was uncomfortable lying down.” -- (WF_WC_M_56, 56, Male, Weifang Weicheng District) -- The pain experience*

*“I have seen those being infected, they are in pain, he/she has herpes on his waist, like the snakes around his waist, and he wants to die”-- (NN_JN_F_70, 70, Female, Nanning Jiangnan District) -- The pain experience of others*

#### *Facilitator 2: Perceived high susceptibility to HZ*

Perception on high susceptibility to HZ infection was related to a positive attitude to HZ vaccine or having been vaccinated, and the factors contributing to this perception included participant age and the past experience of infection.

*“I think that after vaccination my health and resistance to disease will improve, I am old and once the body defense becomes weaker (it is easy to get infected). My husband was also vaccinated, we both were vaccinated.” -- (BJ_DX_F_58, 58, Female, Beijing Daxing District) -- Perception on the elderly being more susceptible to HZ*

*“I had the HZ vaccine. [Interviewer: Have you been infected by HZ?] Yes, I have, but not very serious, and I had been infected five times (before vaccination). The last time herpes were on the waist, and the first time it was on the face, I went to the hospital and received a medicine, and it went away. It's all right. Then 2019 the herpes were on my face, too. It's all right.” -- (WF_WC_M_52, 52, Male, Weifang Weicheng District) -- Repeated infection and susceptibility being high*

#### *Facilitator 3: Advocation by knowledgeable health authority*

Some participants had no clear perception of HZ and the HZ vaccine, but they mentioned that they trusted the recommendation of health professionals and health authorities.

*“I had HZ vaccination. [Interviewer: How did you know about this vaccine?] The doctor recommended it and then I was vaccinated” -- (NN_QX_F_69, 69, Female, Nanning Qingxiu District) -- Recommendation of doctors*

*“This concept (vaccination) was introduced by the COVID-19 pandemic, and many people have only understood it (since COVID-19 vaccine). Vaccination can prevent diseases, the influence of government’s advocate is very important for one to accept or not accept vaccine.” (BJ_CY_F_65, 65, Female, Beijing Chaoyang District) -- The importance of government advocate*

*“[Interviewer: What kinds of factors did you consider whether accept vaccines for your baby?] I don’t know, we just accept what they (community/government policy) arranged for us to be vaccinated.” (NN_JN_F_26, 26, Female, Nanning Jiangnan District) -- The importance of government policy*

#### *Facilitator 4: Financial support for HZ vaccine*

The HZ vaccine is not covered by China’s National Immunization Program, so all HZ vaccination costs are self-paid. The only vaccine on market is the imported Shingrix® vaccine with a high two dose RMB3600 (USD446) price. There were participants mentioning the need for some kind of financial support or subsidies, or to be rich, to facilitate HZ vaccination.

*“A so-called rich friend of mine has been looking for this vaccine. I don't know if he knows how expensive it is and he has to pay for it.” (BJ_CY_F_65, 65, Female, Beijing Chaoyang District) -- Those being able to afford the vaccine are more likely to be vaccinated*

*“[Interviewer: What kinds of vaccines have you taken?] I was vaccinated of all vaccines based on the government’s arrangement, and were never get the vaccines at my own expenses (not supported by government funding)” (NN_JN_F_32, 32, Female, Nanning Jiangnan District) -- The importance of government financial support*

## Discussion

This study applied focus group discussion to collect qualitative data, explored the attitude of urban adults to HZ vaccination and analyzed the barriers and facilitators for HZ vaccine uptake in three Chinese cities. In the process of analysis, the themes on participants’ views towards HZ and HZ vaccination were extracted and grouped based on HBM, including perceived severity and susceptibility to the disease and the perceived benefits and risks of the vaccine. HBM had been used to analyze the predictors of the intent to receive different kinds of vaccines, like COVID-19 [33] and HPV vaccine [34].

Our study found 27.1% of the participants had a willingness to take up the HZ vaccine, which was higher than the 17% in a 2021 survey of the elderly in Shanghai [28], but lower than a 2020 online national survey showing 43% with the intention to HZ vaccination [35]. While previous quantitative research, using participants with different ages and in different regions, is not directly comparable to our focus group approach, both approaches show that the HZ vaccination intention in China was low.

We found that the most prominent barrier to HZ vaccination was the limited knowledge on HZ and the HZ vaccine. People possessing a level of information on health problems and their related treatment methods tended to form a clear view on the risks and benefits of taking vaccination and make rational decisions on whether to vaccinate or not. Several quantitatively studies also found that the level of knowledge on HZ and the HZ vaccine positively influenced the willingness to vaccination [22], frequently positively correlated to people’s education level [15].

The barriers identified in our study decrease the vaccination intention, by reducing the perceived benefits and increasing the perceived risks of getting vaccinated. The low susceptibility, mild symptoms and the curability of HZ, and low cost of treating HZ, meant participants assessed the probability of being infected and, the loss in the quality of life after being infected and financial burden from the infection, were not high enough to motivate the vaccination intention. The perception of the low protection efficacy of the HZ vaccine further reduced the expected benefits from HZ vaccination. The high price of the HZ vaccine, long waiting time to get vaccinated and efforts to locate vaccination sites imposed significant financial and time costs on the vaccination intention, especially when combined with the concerns about the risks and indirect costs from the HZ vaccine. Like other qualitative study, quantitative analyses also found that worries of being infected, confidence in the efficacy of the HZ vaccine, and females were all correlated with a higher willingness to vaccination [17, 21]. There is evidence that females were more risk averse and had lower self-assessed health status level than men [36]. The participants of our study also showed that in female group the proportion with positive attitude to vaccine was a little higher than the proportion in the male group (12/36 in female and 4/23 in male).

The facilitators extracted from the focus group discussions worked to promote participants’ perception of the benefits HZ vaccine uptake. Those who had strong worries about serious pain, a higher vulnerability to HZ and elevated risks from HZ infection, meant the benefits from vaccine uptake were perceived as high. The participants revealed that HZ vaccination information and advocacy by government health officials and health professionals improved participants’ belief on HZ vaccine being both safe and with a high efficacy. Another key finding was the expense of the HZ vaccine was perceived as being significantly higher than the costs of treatment. By directly reducing the cost of HZ vaccination through government or private insurance, HZ vaccine financial support would increase HZ vaccination behavior. Quantitative survey data confirms our finding that higher income was related to a higher vaccination intention, which corresponds to a facilitator in our study [37].

Our study identified novel and previously unrecognized factors shaping HZ barriers and facilitators. The focus group discussions revealed how negative HZ experiences, including those of friends and family, acted as facilitators to HZ vaccination. This finding is consistent with an Italian questionnaire study that included the variable “knowing someone who had suffered from HZ”, which also found a positive relation between HZ experiences and HZ vaccination intention [37]. Consistent with our study, a Danish interview study found knowledge about the disease, personal risk assessment and the recommendations of general practitioners shaped HZ vaccination intention [38]. Our study identified advice from health professionals and government health officials facilitated HZ vaccination uptake. China’s National Immunization Program has established an universal vaccination network covering 14 vaccines, providing an excellent platform to launch a HZ vacation drive. Experiencing over 40 years of a national vaccination program, Chinese residents have a positive attitude towards public health vaccination, advocated and financially supported by the government [39]. Participants mentioned the COVID-19 vaccination movement as a successful government advocated and fully subsidized program, but participants reported that for HZ vaccine they rarely received any HZ recommendations, and sometimes incorrect information, from health workers in community health centers. In the absence of a national HZ vaccination campaign, health professionals had little motivation to promote HZ vaccination and had limited knowledge of the HZ vaccine, which constrained residents’ knowledge of the HZ risks and HZ vaccine benefits. Studies in US, Italy and Denmark found that receiving a recommendation from a healthcare provider was one of the leading factors that influenced willingness to receive the HZ vaccine [40]. Based on the similar findings of this study and other countries’ studies, it is recommended that public health authorities, like hospitals, community health centers expanded the public education and advocacy around HZ and the HZ vaccine. Finally, the HZ vaccine was expensive, so we recommend government subsidies, or health insurance cover, for the HZ vaccine, especially among those vulnerable to HZ.

There are several limitations in this study. Firstly, this study was conducted in three cities with different socio-economic characteristics, generalizing our results to other regions in China and other countries is dependent on comparative context factors, including the HZ vaccine price, national health insurance policy, the population’s health literacy and the payment capacity of residents. Secondly, the focus group discussions were conducted in a primary care setting. Participants in contact with primary health workers might gain some basic knowledge on HZ and the HZ vaccine, which may over-estimate the knowledge level of the general population. Thirdly, participants from different age groups were in the same focus group, which potentially caused the views of one age group being influenced by another age group.

## Conclusions

Our focus group discussions revealed the low proportion of participants with a willingness to HZ vaccination. Perceived vulnerability to HZ and fear of HZ pain were the strongest facilitators competing with limited or incorrect conception on the prevalence, risk factors, susceptibility, symptoms, prevention and treatment methods as barriers to HZ vaccination. We found that advocacy by health workers or government health officials, and individuals’ financial capacity or government subsidies to HZ vaccination, could attenuate the barriers to HZ vaccine uptake. We recommend HZ education and advocacy by health workers and government health officials to address the populations limited HZ knowledge and HZ misconceptions, and for the government, or health insurance providers, to pay or subsidize the high costs of HZ vaccination. Future studies on HZ vaccination should focus on monitoring and evaluating the immunization promotion strategies targeting the barriers found in this study.

## Data Availability

Data can be obtained from the corresponding author with reasonable request.

## Abbreviations

HZ: Herpes Zoster
LMICs: Low- and middle-income countries
